# Students’ perspectives on the use of digital versus conventional dental impression techniques in orthodontics

**DOI:** 10.1186/s12909-019-1512-3

**Published:** 2019-03-12

**Authors:** Timm C. Schott, Rahima Arsalan, Katja Weimer

**Affiliations:** 1 Department of Orthodontics and Orofacial Orthopedics, University Hospital of Dentistry, Oral Medicine, and Maxillofacial Surgery, Eberhard Karls University Tübingen, Osianderstr. 2-8, 72076 Tübingen, Germany; 2 Department of Psychosomatic Medicine and Psychotherapy, University Hospital Tübingen, Tübingen, Germany; 3 Department of Psychosomatic Medicine and Psychotherapy, Ulm University Medical Center, Ulm, Germany

**Keywords:** Dental student, Digital impressions, Alginate impressions, Training, Intraoral scanner

## Abstract

**Background:**

Despite the increasing use of digital impressions in orthodontics, this technique does not usually form part of the learning objectives in dental training. The aim of this study was to determine how students assess the user-friendliness of intraoral scanners compared to a conventional impression technique after a theoretical and practical teaching module.

**Methods:**

Thirty-one dental students in their seventh semester (4th year) received and conducted digital (3 M, St. Paul, NM) and conventional (alginate) impressions from: (i) the dentist’s perspective, and (ii) the patient’s perspective. Each student completed four questionnaires to evaluate: (i) the user-friendliness of intraoral scanning, and (ii) intraoral scanning compared to the conventional method.

**Results:**

Thirty (97%) students had not previously performed digital impressions. Twenty-four (77%) students were overall “very” or “rather” satisfied with the handling of the intraoral scanning method, and 18 (58%) preferred digital to alginate impressions from the dentist’s perspective. From the “patient’s” perspective, the students did not report any significant differences between the two methods. However, the impression tray in conventional impressions reduced “patient” comfort significantly more than the camera in digital impressions (Z = − 3.496, *p* < 0.001).

**Conclusions:**

Dental students were able to practice both conventional alginate and modern digital impressions without prior knowledge of intraoral impression techniques after basic training and an introduction from dentists. Students reported a preference for the digital technique. Implementing digital intraoral impressions into undergraduate training is recommended to familiarise students with this rapidly developing digital technique at an early stage.

## Background

Conventional impressions usually accurately represent the intraoral environment for the manufacture of orthodontic appliances (dental aligners, removable (retention) devices) or study casts for orthodontic treatment planning. Various impression methods and materials, especially alginate and elastomers, are available for this purpose. Good impressions form the basis for precise, high-quality treatment appliances [1–5]. Therefore, impressions aim to depict the intraoral environment with as much detail and dimensional accuracy as possible. Imprecise impressions compromise the fit of orthodontic appliances e.g., for removable appliances like aligners or indirect bonding techniques [6].

Digital technology now offers an alternative approach to conventional impressions for appliance fitting and manufacture, and a number of commercial intraoral scanners are available for digital impressions. All the scanners are based on optical imaging, although the techniques (and thus practical handling) differ. Several studies have shown that intraoral scanners outperform conventional impression techniques [7, 8]. In addition, dental hygienists have started to favour digital impressions as a result of practical training with intraoral scanners [9]. The intraoral scanner, when combined with high-performance computing and automated protocols, forms the basis for many applications such as digital or printed study casts or digital set-ups for individualized appliances.

In contrast to the original application of computer-aided design/computer-aided manufacturing (CAD/CAM) technologies in prosthetics, orthodontics places different demands on these devices. Intraoral scanners must not only accurately represent smaller areas such as individual teeth or tooth groups, but also entire dental arches. This creates new challenges [10].

Despite the increasing use of digital impressions in orthodontics, this new technique does not yet form part of the learning objectives for dental undergraduates. Given the widespread use of digital impressions in practice, this technology must be taught as a modern alternative to conventional impression techniques, not least because it has recently been shown that the majority of dentistry students will go on to use digital impressions in practice [11].

Here we aimed to determine how students assess the user-friendliness of intraoral scanners compared to conventional impressions after a theoretical and practical teaching module. Students, the majority of whom had neither worked with intraoral scanners nor with conventional orthodontic impressions, gained initial experience with intraoral scanning and could draw a direct comparison with conventional impressions - both from the practitioner’s and patient’s perspectives - by performing both impression methods. Finally, students were asked to rate and provide feedback on the teaching module to assess its efficacy.

## Methods

### Participants

The study population consisted of 31 dental students in their seventh semester (corresponds to the 4th year out of five) at the University’s Medical Centre for Dentistry, Oral Medicine and Orthodontics at University Hospital, Tübingen, Germany. All participation was voluntary. Each subject experienced conventional and digital impressions from (i) the dentist’s perspective and (ii) the patient’s perspective.

Students received a detailed introduction to both methods by means of training courses and supervision from experienced tutors while carrying out the impressions, with each induction one week apart. A dentist with broad experience in handling intraoral scanners led the introduction for the digital method, a fully trained dentist also introduced the conventional method including bite registration with bite wax.

### Digital impressions

The 3 M True Definition intraoral scanner (3 M, St. Paul, NM) was used. This intraoral scanner uses ‘wavefront sampling’ as a passive triangulation method. Before the actual scanning process, a thin titanium oxide reflective powder is applied to the tooth surfaces, the powder acting as randomly distributed landmarks for the optical impression system. Several cameras within the scanner head capture the objects (the tooth surfaces) simultaneously from different perspectives, similar to stereopsis of the human eye. For complete impressions, the scanner head must be guided along all tooth surfaces. The intraoral scanner generates monochrome, digital data records, which are then visualised as a digital model based on video sequences [12–14].

### Performing digital impressions

Following completion of a pre-introduction questionnaire (No. 1), the tutor started with a one-hour presentation about the background, development and clinical use of the digital scanning technique, followed by the introduction of the technique of performing digital impressions including bite tacking with an intraoral scanner. Afterwards, the dentist performed a live demonstration on a phantom head. Guided by three dentists, the students then carried out impressions at three stations, each equipped with an intraoral scanner. Three students formed a team, with each team member experiencing being a patient and a dentist to allow each student to evaluate digital impressions from different perspectives. Before each performance, the students received another live demonstration in a small group of three at the respective dentist’s chair, followed by their own performing digital impressions of both jaws including bite taking. Data were stored and the digital model created only after the leading dentist had rated the impression as clinically acceptable.

### Conventional impressions and bite registration method

At the beginning of the study, students have only had one upper and one lower jaw impression in their training during first semester (1st year), so one week before performing the digital impressions, the students also received an introduction to the conventional impression method in orthodontics including bite registration with bite wax before performing conventional impressions in groups using irreversible hydrocolloid alginate (Kaniedenta, Herford, Germany) according to the manufacturer’s instructions with standard metal trays. Each student carried out an upper and a lower jaw impression on his or her fellow student, so each student also had their own impressions taken. In addition, each student created a bite registration in centric occlusion with bite wax. As all students had a good centric occlusion, the focus of the training was not on bite registration. A tutor (dentist) then examined the impressions and bite wax registration for their clinical usability before they could be poured out.

### Questionnaires

Questionnaire compilation was based on a comprehensive literature review. Closed questions were chosen with the following answers: “do not agree at all”, “rather disagree”, “somewhat agree”, and “fully agree”.

Each student completed four questionnaires to evaluate the user-friendliness of intraoral scanning as well as intraoral scanning compared to the conventional method. The first section of the four questionnaires (No. 1–4) consisted of the creation of an individual code that ensured anonymity: the first two letters of the mother’s first name, the mother’s month of birth, the first two letters in the father’s first name, and the father’s month of birth.

Questionnaire No. 1 was administered before the students were instructed on digital impression-taking. At the beginning of the teaching session, the students completed this pre-induction questionnaire that asked questions about: a) previous experience with intraoral scanners, and b) the expected user-friendliness of digital impressions. The students were not asked about their previous experience with conventional impressions, as it was known that all students already had some “in vitro” and one “in vivo” experience with conventional impressions in their training.

After the introductory sessions on digital and conventional methods and practical methods of making impressions, both methods were evaluated from the patient’s perspective using two questionnaires (No. 2 and No. 3) containing seven questions about: well-being, comfort, pain intensity, possible breathing problems, taste, and possible nausea.

Questionnaire No. 4 enquired about the user-friendliness of intraoral scanning from the practitioner’s point of view, with questions on: duration, handling of the scanner, use of the powder, software operation, volume, model presentation, tolerance to the powder, and overall satisfaction. In addition, questions were posed about the differences between conventional and digital impressions.

This teaching module was evaluated by the students at the end of the semester using the institution’s online standardized evaluation form. The evaluation form asked about the clarity and transparency of educational objectives and contents, personal interest in the topic, and if teachers encouraged critical examination of the topic. All ratings were given on a five-point Likert scale from “fully agree” to “not agree at all”. Finally, they gave an overall rating about the module according to German school marks with 1 = “very good”, 2 = “good”, 3 = “satisfactory”, 4 = “fair”, and 5 = “insufficient”.

### Statistical analysis

Statistical analyses were carried out in IBM SPSS Statistics for Windows, version 19.0 (IBM Corp., Armonk, NY). To ensure data quality, data were entered twice and checked for input errors and plausibility. Missing data were treated as “missing values” and were not taken into account in the relevant analysis. Results are reported as means and standard deviations (M ± SD), as absolute and percentage frequencies, or as medians (Md) and interquartile ranges (25th - 75th percentile, IQR) where appropriate. The comparison between digital and conventional impressions was analysed using the Wilcoxon Rank test for dependent samples and the Z-values are reported. The significance level was set to α = 0.05.

For Wilcoxon tests, an a priori sample size calculation revealed an optimal n of 35 students to detect a medium effect size of dz. = 0.5 with α = 0.05 and power = 0.80 (calculated with G*Power Version 3.1.9.2) [15]. However, this course comprised only 31 students and could not be enlarged for organisational reasons. Therefore, we assessed post hoc-achieved effect sizes and powers of the two significant results with *n* = 31 students. These calculations revealed an achieved effect size of dz. = 0.82 and power = 0.99 for the question about “patient” comfort, and dz. = 0.49 and power = 0.73 for the question about breathing difficulties (see Table 2). Because of the large effect sizes, the small sample size was sufficient for data analyses.

## Results

### Students’ impressions of digital impressions

The results are summarised in Table 1. The students’ average age was 24.7 ± 2.5 years (range 21 to 31 years); 22 were female and 9 were male. Twenty-eight out of 31 students (90.3%) had not previously worked with an intraoral scanner; three (9.7%) indicated that they had had some previous experience. Thirty students (96.8%) had not yet performed digital impressions themselves and were not familiar with an intraoral scanner, while only one reported that he or she had had some previous experience. In terms of expected user-friendliness, 28 (90.3%) students expected the digital impression to be (rather) user friendly.

**Table 1:** Students’ evaluation of user-friendliness of digital impressions. Response scale: 1 = “Do not agree at all”, 2 = “Rather don’t agree”, 3 = “Agree” to 4 = “Fully agree”. Md, median; IQR, interquartile range

Item	Md	IQR
The time required to perform digital impression is satisfactory	3.0	2.0–3.0
I feel that that digital impression is too time-consuming	2.0	2.0–3.0
I am very satisfied with operating the intraoral camera	3.0	2.0–3.0
The hand piece is too big for intraoral use	2.0	2.0–3.0
The use of the powder was straightforward and fast	3.0	3.0–4.0
I experienced the use of the powder as cumbersome	2.0	1.0–3.0
I dealt well with operating the programme software	3.0	3.0–4.0
Operation through the program was very complicated and time-consuming	1.0	1.0–2.0
I am satisfied with the volume of the intraoral scanner	4.0	3.75–4.0
I am satisfied with the presentation of the digital model on the monitor	4.0	3.0–4.0
I am satisfied with the oral tolerance towards the handpiece	3.0	3.0–4.0
I am satisfied with the oral tolerance towards the powder	3.0	3.0–4.0

Upon completion of training and independent application of the intraoral scanner, 22 (71.0%) students rated digital impressions as rather user-friendly. Thirteen (41.9%) considered the implementation to be rather cumbersome, and 14 (45.2%) students considered it to be difficult.

Overall, the assessment of digital impressions with regard to time taken, camera handling, use of powder, software operation, device volume, digital model presentation, and intraoral irritations was favourable. Twenty-four (77.4%) students indicated that they were overall very or rather satisfied with the handling of the intraoral scanning method.

### Comparison of digital and conventional impressions

All students needed to produce at least two conventional alginate impressions without exception due to different errors arising during the first impression. Errors included pressed in points and incomplete impressions (often due to the absence of the second molars), bubbles, and premature curing of the impression material. Eighteen (58.1%) and 21 (67.7%) students reported that conventional impressions with alginate were (rather) easier or faster than digital impressions, respectively. Nevertheless, 18 students (58.1%) still preferred digital to alginate impressions.

From the patient’s point of view, students reported both impression techniques to be equally satisfactory and tended to disagree with the negative side-effects of impression techniques. However, the impression tray in conventional impressions reduced “patient” comfort significantly more than the camera did in digital impressions (Z = − 3.496, *p* < 0.001). Alginate impressions were more likely to lead to breathing difficulties than the digital impressions (Z = − 2558, *p* = 0.011) (Table 2).

**Table 2:** Comparative evaluation of alginate and digital impressions from the patient’s perspective (for response scale, see Table 1). Md, median; IQR, interquartile range

Item	Alginate Md [IQR]	Intraoral scanner Md [IQR]	Statistics Z, *p*-value
I felt comfortable during the impression with alginate/the intraoral scanner	3.0 [2.0–3.0]	3.0 [2.0–3.0]	Z = − 0.673, *p* = 0.501
I experienced the impression with alginate/the intraoral scanner as uncomfortable	2.0 [2.0–3.0]	2.0 [1.0–3.0]	Z = − 0.533, *p* = 0.594
During alginate impression/digital impression, the impression tray/the camera limited my comfort	3.0 [3.0–4.0]	2.0 [1.0–3.0]	Z = − 3.496, *p* < 0.001
I experienced the alginate impression/ digital impression as painful	1.0 [1.0–2.0]	1.0 [1.0–2.0]	Z = − 0.778, *p* = 0.436
During the alginate impression/digital impression I experienced breathing difficulties	2.0 [1.0–3.0]	1.0 [1.0–2.0]	Z = − 2.558, *p* = 0.011
I experienced the taste of the impression material/powder as unpleasant	2.0 [1.0–2.0]	1.0 [1.0–2.0]	Z = − 0.762, *p* = 0.446
During alginate impression/digital impression, I complained about nausea	1.0 [1.0–1.0]	1.0 [1.0–2.0]	Z = − 0.516, *p* = 0.606

### Evaluation of the teaching module

Students reported that the educational objectives were clearly defined (1.4 ± 0.6), the content of the teaching module was transparent (1.4 ± 0.6), and knowledge was imparted understandably (1.6 ± 0.6). The teaching unit promoted their interest in the topic (1.7 ± 0.8), and teachers mostly encouraged critical examination of the topic (2.2 ± 1.0). Finally, students graded the teaching unit with a “very good” to “good” mark (1.6 ± 0.6).

## Discussion

Here we studied dental students’ experiences of practitioner’s and patient’s perspectives of intraoral scanning after being given an introductory module on conventional and digital impression methods. We show that the same time effort was required to instruct and train inexperienced students in conventional impressions with elastomers as modern digital scanning with an intraoral scanner. After the students had practised both techniques in the active role of “practitioner” and in the passive role of “patient”, the majority of students reported a general preference for using the digital technique in the future, despite slight reservations with the digital impression technique.

The students rated the user-friendliness of the intraoral scanner as positive to very positive, most likely due to a lower error rate in producing digital impressions. During the digital method, students can concurrently use a screen to see if they are scanning correctly, with possible errors flagged in real-time. The appearance of, for example, “red arrows” (which indicate an inaccurately detected scan area) or colourless “holes” make it possible to identify insufficiently scanned data in the corresponding section. Ultimately, these functions can be used to immediately determine whether sufficient scanned data has been collected and, during scanning, one is informed of any necessary corrections so that changes are made in real-time.

For conventional alginate impressions, it is necessary to wait until the imprint has hardened prior to removal from the patient’s mouth. The success of the impression can only be determined afterwards, and subsequent processing is not possible. According to Yuzbasiouglu et al. [16], the accuracy of the final master cast depends on numerous factors including the water/powder ratio, vacuum versus hand mixing [17–19], and the type of dental stone and its compatibility with impression materials [20]. These possible sources of error are mitigated if the “digital working model” is directly obtained from the intraoral scan [16].

A slight majority of students held the view that, from the practitioner’s point of view, the conventional alginate impression was (rather) easier or faster to perform than intraoral scanning with the True Definition scanner. This may be in part due to the True Definition workflow, since a thin powdering of the tooth surfaces is needed before performing the actual impression with this system. In this study, however, the individual times taken to execute the respective methods were not determined because the time required to perform an impression mainly depends on the experience and routine of the practitioner. Therefore, considerable individual time differences might be expected in this inexperienced group. Previous studies have shown that the time required for clinically acceptable intraoral scans is reduced with increasing experience and with the time taken to execute a digital impression [21].

We did not perform a full health economics analysis. For digital impressions, acquiring a scanner has high investment costs that do not apply to conventional impressions. However, a considerable advantage of digital impressions is that digital documentation is logistically much easier to implement than with conventional impressions using plaster models. If several impressions are required successively to document the course of therapy, digital impressions provide a decisive advantage compared to conventional approaches. Changes in the intraoral environment are easily represented by digitally overlaying digital impressions from different time points.

From the patient’s perspective, the students did not report any significant differences between the two impression methods. The students regarded the use of impression trays in the conventional technique as a limitation to comfort compared to the camera used during digital scanning. It can reasonably be assumed that children, adolescents, and adults experience real conventional or digital impression differently and distinctly than dental students, who have a professional understanding of the impression-taking after completing a teaching module. The use of different scanners and protocols will also introduce variability in subjective assessments of these different procedures.

Overall, students rated all aspects of this teaching module as very good to good. Therefore, implementing this module into the general dental curriculum should be considered.

## Conclusions

Dental students could produce both conventional alginate and modern digital impressions successfully without prior knowledge of intraoral impression techniques after basic training and an introduction from dentists. The students did not notice any significant differences between the methods, either from the “practitioner’s” or the “patient’s” perspectives. Students reported a preference for the digital technique. Implementing digital intraoral impressions into undergraduate training is recommended to familiarise students with this rapidly developing digital technique at an early stage in order to be able to practice it in modern orthodontics.

## Abbreviations

CAD/CAM: Computer-aided design/computer-aided manufacturing
